# Axl receptor tyrosine kinase is up-regulated in metformin resistant prostate cancer cells

**DOI:** 10.18632/oncotarget.4148

**Published:** 2015-05-15

**Authors:** Nitu Bansal, Prasun J. Mishra, Mark Stein, Robert S. DiPaola, Joseph R. Bertino

**Affiliations:** ^1^ Rutgers Cancer Institute of New Jersey, Rutgers The State University of New Jersey, New Brunswick, NJ, USA; ^2^ Department of Biochemical and Cellular Pharmacology, Genentech, South San Fransisco, CA, USA

**Keywords:** Axl receptor tyrosine kinase, metformin, prostate cancer, Axl and drug resistance

## Abstract

Recent epidemiological studies showed that metformin, a widely used anti-diabetic drug might prevent certain cancers. Metformin also has an anti-proliferative effect in preclinical studies of both hematologic malignancies as well as solid cancers and clinical studies testing metformin as an anti-cancer drug are in progress. However, all cancer types do not respond to metformin with the same effectiveness or acquire resistance. To understand the mechanism of acquired resistance and possibly its mechanism of action as an anti-proliferative agent, we developed metformin resistant LNCaP prostate cancer cells. Metformin resistant LNCaP cells had an increased proliferation rate, increased migration and invasion ability as compared to the parental cells, and expressed markers of epithelial-mesenchymal transition (EMT). A detailed gene expression microarray comparing the resistant cells to the wild type cells revealed that *Edil2, Ereg, Axl, Anax2, CD44 and Anax3* were the top up-regulated genes and calbindin *2 and TPTE* (transmembrane phosphatase with tensin homology) and *IGF1R* were down regulated. We focused on Axl, a receptor tyrosine kinase that has been shown to be up regulated in several drug resistance cancers. Here, we show that the metformin resistant cell line as well as castrate resistant cell lines that over express Axl were more resistant to metformin, as well as to taxotere compared to androgen sensitive LNCaP and CWR22 cells that do not overexpress Axl. Forced overexpression of Axl in LNCaP cells decreased metformin and taxotere sensitivity and knockdown of Axl in resistant cells increased sensitivity to these drugs. Inhibition of Axl activity by R428, a small molecule Axl kinase inhibitor, sensitized metformin resistant cells that overexpressed Axl to metformin. Inhibitors of Axl may enhance tumor responses to metformin and other chemotherapy in cancers that over express Axl.

## INTRODUCTION

Metformin belongs to the biguanide class of compounds and is widely used in the treatment of type II diabetes. Population studies have shown that type II diabetic patients treated with metformin have a lower incidence of certain cancers as compared to non-metformin users [1]. Although a recent study concluded that the use of metformin after a prostate cancer diagnosis was not associated with an overall decreased risk of cancer-specific and all-cause mortality [2], in another study conducted on a larger number of patients the authors concluded that increased duration of metformin exposure after prostate cancer diagnosis was associated with decrease in prostate cancer-specific mortality among diabetic patients [3].

Although the complete mechanism of action of metformin against cancer is not known, it has been suggested that metformin inhibits cancer growth by a similar mechanism to decreasing blood glucose levels in diabetic patients [4]. Metformin treatment inhibits hepatic glucose output and improves insulin sensitivity, lowers circulating insulin levels and activates AMP-activated protein kinase (AMPK) that inhibits glucose production by the liver and improves glucose uptake by muscle [5]. Activated AMPK inhibits mTOR, a kinase that regulates cell growth, cell proliferation, cell motility, cell survival, protein synthesis, and transcription. The activation of AMPK by metformin is dependent of the AMP/ATP ratio [6]. However, there are other studies suggesting that metformin can also inhibit gluconeogenesis in an AMPK independent pathway by altering the AMP/ATP ratio [6, 7].

Pre-clinical experiments have indicated that metformin can have an anti-proliferative effect in several solid cancers that include colon, breast, pancreatic and prostate tumors, as well as in hematologic malignancies [8]. Many clinical trials are currently under way to investigate metformin's potential to prevent and treat different cancers. If metformin has anticancer activity in the clinic, intrinsic or acquired resistance to this drug would be expected.

To understand the mechanism of acquired resistance, and possibly understand its mechanism of action as an anticancer drug, we generated metformin resistant LNCaP cells by exposing the cells continuously to metformin until a resistant subline was generated, and performed a microarray gene analysis comparing the resistant cells to the parent cells. A striking finding was that Axl receptor tyrosine kinase was up regulated 34 fold in metformin resistant (MetR) cells. Knock down of Axl in the MetR cells sensitized the cells to metformin as and overexpression of Axl in parenteral LNCaP cells, sensitive to metformin, made the cells resistant to metformin. Moreover, knock down of Axl in Du145 cells that inherently over express Axl sensitized the cells to metformin. R428, a novel small molecule inhibitor of Axl in early clinical trials for the treatment of patients with cancer, is shown to be a potent inhibitor of prostate cancer cells and the combination of R428 with metformin in Axl over expressing cells showed additive to synergistic cell kill. Importantly, MetR cells showed changes associated with EMT, that include up regulation of twist and vimentin, highlighting the role of Axl mediated EMT in drug resistance.

## RESULTS

### Metformin inhibits prostate cancer cell growth, activates AMP kinase and inhibits AKT and cyclin D1

**Figure 1:**
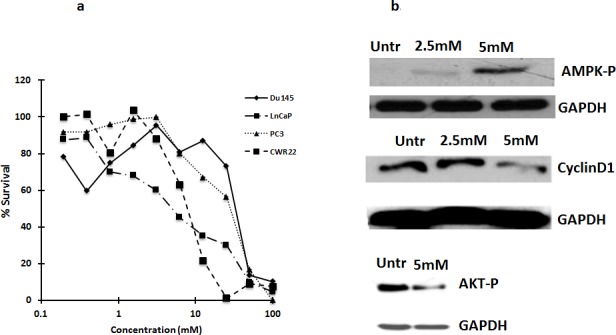
**a. Metformin is cytotoxic to several prostate cancer cells:** Castrate resistant prostate cancer cells Du145, PC3, are less sensitive to metformin as compared to androgen dependent prostate cancer cells, CWR22 and LNCaP. Cells were treated with metformin and after 72 h, MTS reagent was added and absorbance was measured at 490nm. Percent survival was calculated by normalizing the values to untreated. Data are represented as mean +/−. **b.** Metformin inhibits activation of AMPK and reduces Cyclin D1 levels: Western blot analysis showing the expression of cyclin D1 and phospho-AMPK in LNCaP cells treated with metformin for 72 h. 50ug of total protein was loaded on SDS page gel and blotted with anti-cyclin D1 and P-AMPK antibodies.

To determine if metformin is cytotoxic to prostate cancer cells, LNCaP, Du145, PC3 and CWR22 cells were treated with metformin at different concentrations ranging from 0.75mM to 50mM for 72 h. MTS assay was done post 72 hours treatment. As shown in Figure 1a, LNCaP and CWR22 cells were almost ten-fold more sensitive to metformin than the castrate resistant PC3 or DU145 cells (IC50 concentrations at ~5mM vs of 50mM).

As earlier studies demonstrated that metformin inhibits gluconeogenesis by activating AMP kinase [9], we tested the effect of metformin on AMP kinase in LNCaP cells. Metformin activated AMP kinase, inhibited AKT phosphorylation and decreased cyclin D1 levels in LNCaP cells (Figure 1b). Metformin has been shown to inhibit cell proliferation by blocking the cells in G0/G1 phase of cell cycle associated with reduced levels of cyclin D1 [9].

### Establishment of metformin resistant LNCaP cells

**Figure 2:**
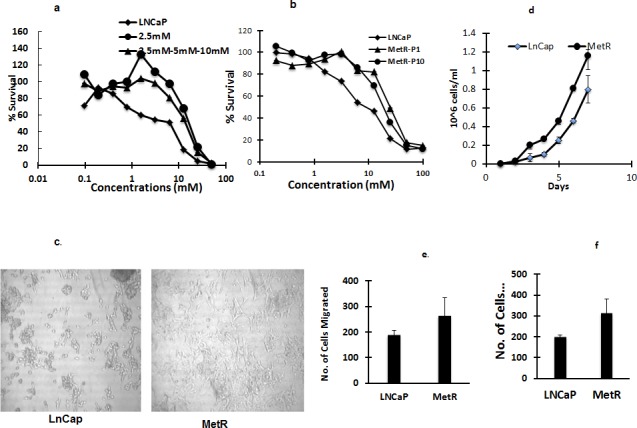
**a, b. Establishment of metformin resistant LNCaP cells:** Metformin resistant cells are 4 fold resistant to metformin compared to LNCaP cells. LNCaP cells and MetR cells established by either continuous exposure of 2.5mM or by exposing cells with increasing concentrations of metformin (2.5mM-5mM-10mM) were treated with metformin for 72 h and MTS assay was done to measure percent survival and determine the IC50 concentrations. **b.** MetR cells were cultured and grown without metformin. Passage 10 (P10) cells were treated with metformin and MTS assay was performed to determine the stability of resistance. **c.** Morphology of MetR cells is mesenchymal like: Images of LNCaP and MetR cells were taken using bright field microscopy. **d**, **e** and **f.** MetR cells have increased proliferation, migration and invasion rates: 10,000 LNCaP and MetR cells were washed and suspended in serum free media and plated in transwell inserts in 24 well plates. For **f.** the transwell inserts were coated with 100ug/ml of matrigel. Lower chamber had 10% FBS containing regular media. 24 h later the cells in the inserts were washed and lower chamber was stained with crystal violet. Stained cells were counted and plotted. The experiments were done in triplicates and three times independently. Data are represented as +/− SD).

To understand the mechanism of action of metformin and inherent and acquired resistance to metformin in prostate cancer cells, we generated LNCaP cells resistant to metformin by treating LNCaP cells repeatedly (over 10 generations) with a fixed 2.5 mM (IC50) concentration of metformin, allowing cells to regrow between treatments, to simulate clinical treatments, In addition we also treated LNCaP cells with increasing concentrations of metformin (2.5mM-5mM-10mM). Similar levels of resistance (4-fold) were obtained with each method. Further increases in drug concentration or additional exposures at the IC50 concentration did not increase the level of resistance. To determine if the acquired resistance to metformin was stable, resistant cells were grown in media without metformin. Resistant cells (MetR) grown without metformin were resistant to metformin even after 10 passages (Figure 2b). Morphology of the MetR cells differed from the parental cells. The MetR cells had spread out filopodias and a flattened morphology (Figure 2c), as compared to the sensitive cells. Besides having a distinct morphology, MetR cells also had increased proliferation, increased migration and increased invasion rates (1.5 fold) when compared to parental LNCaP cells (Figure 2d, 2e, 2f).

### Microarray gene expression analysis of LNCaP cells and the MetR cells

**Figure 3:**
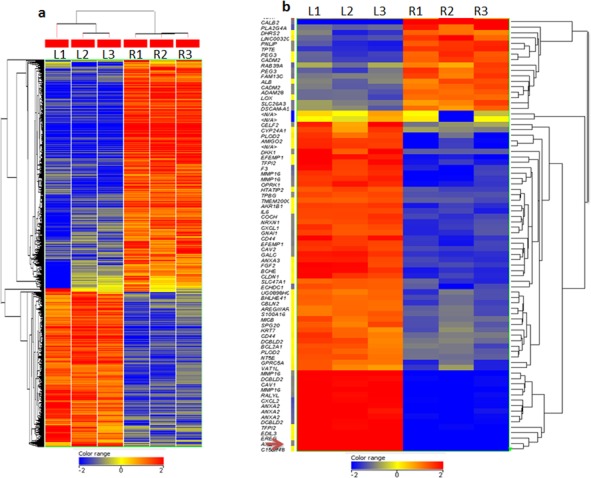
**a, b. Gene expression analysis of LNCaP and MetR cells:** Total RNA was extracted from LNCaP and MetR cells and microarray analysis was done in triplicate (see methods). **a.** Global gene expression analysis data (2 fold and over) is presented demonstrating gene clusters differentially expressed in resistant cells (R) as compared to parental LNCaP cells (L).red: genes overexpressed, blue, genes underexpressed. **b.** Heat map presents top genes (6 fold and over) upregulated and downregulated in resistant cell lines (R) as compared to parental LNCaP cells (L).

A microarray gene expression analysis of LNCaP cells and MetR resistant cells was performed to find changes associated with resistance. Global gene expression analysis data (2 fold and over) is presented in Figure 3a, demonstrating gene clusters differentially expressed in resistant cell lines (R) as compared to parental LNCaP cells (L). Figure 3b shows the heat map of the top list of genes (6 fold and over) up-regulated and down-regulated in resistant cell lines (R) as compared to parental LNCaP cells (L). *Edil3, Ereg* and *Axl* were the top 3 up-regulated genes (52 to 30 fold). The list of 200 genes up regulated and down regulated in the MetR cells as compared to parenteral LNCaP cells is shown in Supplemental Table 1. *Edil3* (epidermal growth factor-like repeats and discoidin I-like domains 3) is also referred to as Del-1 and integrin-binding DEL1. This is a 52 kDa extracellular matrix protein which is expressed by endothelial tissues during embryonic vascular development [10] Its role in cancer and in drug resistance is not widely studied, however a few studies indicate that Edil-3 overexpression relates to poor prognosis in hepatocellular carcinoma [11]. Also, when down-regulated in colon cancer cells, it inhibits the growth and proliferation of cells [12]. Ereg (Epiregulin) is a member of the EGF family and can function as a ligand for EGFR as well as a ligand for most of ERBB (v-erb-b2 oncogene homolog) receptors [13]. Increased expression of Epiregulin has been shown to be a biomarker for several cancers [13] Axl belongs to the TAM (Tyro-3, Axl and Mer) family of receptor tyrosine kinases [14]. Gas6 binds to Axl and activation of Axl increases proliferation and inhibits apoptosis. Increased expression of Axl has been shown in several drug resistant cancers, e.g., leukemia and lung cancer [15, 16]. *Anxa2* and *Anxa3 (Annexin A2 and Annexin A3)* were also up regulated in the array. CD44, a cancer stem cell factor was also up regulated in the resistant cells. *Calbindin2* or *calretinin* and TPTE (transmembrane phosphatase with tensin homology) were down-regulated in the MetR cells.

### Axl receptor tyrosine kinase

**Figure 4:**
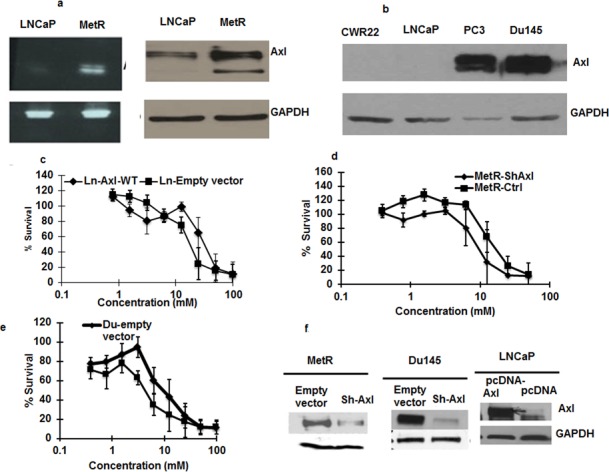
**a. Axl is overexpressed in MetR cells:** RNA and protein was extracted from parenteral LNCaP and MetR cells. RT-PCR analysis using Axl primers were done to detect the presence of Axl mRNA in the cells (left panel). Western blotting with anti-Axl antibody was done with 50ug of total protein loaded on SDS page gel (right panel) **b.** Axl expression differ in different prostate cancer cells: 50ug of total protein from Du145 cells, PC3 cells, LNCaP and CWR22 were loaded on SDS page gel and western blotting was done with anti-Axl antibodies to detect the levels of Axl protein. **c.** Axl overexpression render LNCaP cells less sensitive to metformin: LNCaP cells were transfected with Axl cDNA using lipofectamine 2000 in 96 well plates. 24 h after transfections, cells were treated with metformin. Control cells transfected with empty vector were also treated with metformin and 48 h later, MTS reagent was added and cell survival was measured. **d**, **e.** Axl knock down in MetR and Du145 cells sensitizes the cells to metformin: MetR cells were transiently transfected with si-RNA oligos for Axl with lipofectamine 2000. 24 h after transfection metformin was added. Control MetR cells transfected with scrambled si-oligos were also treated with metformin. 48 h after treatment MTS reagent was added and percent cells survival was measured. **e.** Axl was knocked down in Du145 cells (expressing highl levels of Axl) with ShRNA against Axl by transfection and stable Axl knock down Axl cells were selected with puromycin. Control Du145 cells were transfected with empty lentiviral construct. Both sets of cells were treated with metformin and cytotoxicity was measured as mentioned above. The experiment was done in triplicates and repeated 3 times (data are represented as mean +/− SEM). **f.** Western blot showing Axl overexpression and Axl knock down in LNCaP, MetR and Du145 cells: A fraction of cells that were used in figure **c**, **d** and **e.** were harvested and western blot was done to confirm the expression of Axl in different cell sets.

Axl overexpression has been shown to be present in imatinib resistant leukemia cells and erlotinib resistant non-small cell lung cancer cells [15, 17]. As the mRNA array showed that Axl expression was up regulated 32 fold, we confirmed the high level of expression of mRNA and protein by RT-PCR and Western blot respectively (Figure 4a). Axl is 130 KDa protein, and cleavage at the extracellular domain releases a 85 KDa protein in the media [18]. This 85KDa protein band was found present in MetR cells. Of Interest, Du145 and PC3 cells that are inherently less sensitive to metformin have higher levels of Axl whereas LNCaP and CWR22 cells that are more sensitive to metformin have low expression of Axl (Figure 4b). Therefore to determine if Axl up regulation is associated with resistance to metformin, we overexpressed Axl in parenteral LNCaP cells, and knocked-down Axl in MetR cells. We transiently transfected LNCaP cells with wild type Axl plasmid for 48 h and treated the cells with metformin for 48 hours. As shown in Figure 4c, overexpression of Axl in LNCaP cells sensitized the cells to metformin, while knock down of Axl in MetR cells sensitized the cells to metformin (Figure 4d). Stable knockdown of Axl in Du145 cells, the cell line inherently resistant to metformin, also sensitized the cells to metformin (Figure 4e).

**Figure 5:**
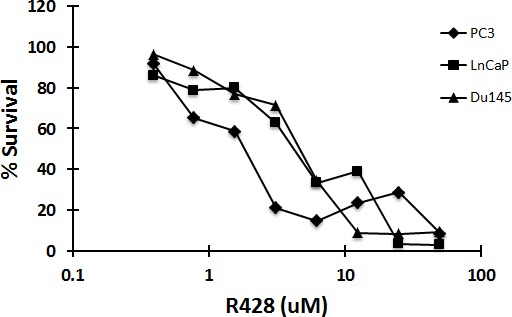
The small molecule inhibitor of Axl, R428, is cytotoxic to prostate cancer cells at low IC50 concentrations Du145, LNCaP and PC3 cells were plated in 96 well plates. Cells were treated with R428 at varying concentrations. After 72 h, MTS reagent was added and absorbance was measured at 490nm. Percent survival was calculated by normalizing the data with untreated values.

We also examined the effect of R428, a selective small molecule inhibitor of Axl [19] alone and in combination in the prostate cell lines. As shown in Figure 5, R428 was cytotoxic to Du145, PC3 and LNCaP cells with IC50 concentrations in the low micromolar range, and when metformin was combined with R428 in DU145 cells there was borderline synergistic cell kill at high drug concentrations (Table 1a). In MetR cells, treatment with R428 resulted in in additive cell kill when combined with metformin (Table 1b). In Du145 cells, R428 and metformin were synergetic at EC50, EC75 and EC90 concentrations whereas in MetR cells, R428 and metformin were synergetic only at EC90 concentrations.

**Table 1a:** Combination Index for metformin and R428 in Du145 cells (Combination index values (CI) values were calculated using CalcuSyn software CI>1 antagonism, CI=1, additive, CI<1, synergy [41])

Drug	CI values at ED50	CI values at ED75	CI values at ED90
R428 (alone)	N/A	N/A	N/A
Metformin (alone)	N/A	N/A	N/A
R428 and metformin (1:5000)	0.91	0.85	0.81

**Table 1b:** Combination Index for metformin and R428 in MetR cells (Combination index values (CI) values were calculated using CalcuSyn software CI>1 antagonism, CI=1, additive, CI<1, synergy [41])

Drug	CI values at ED50	CI values at ED75	CI values at ED90
R428 (alone)	N/A	N/A	N/A
Metformin (alone)	N/A	N/A	N/A
R428 and metformin (1:5000)	1.16	1.00	0.87

### Mechanism of action of Axl in metformin resistance

**Figure 6:**
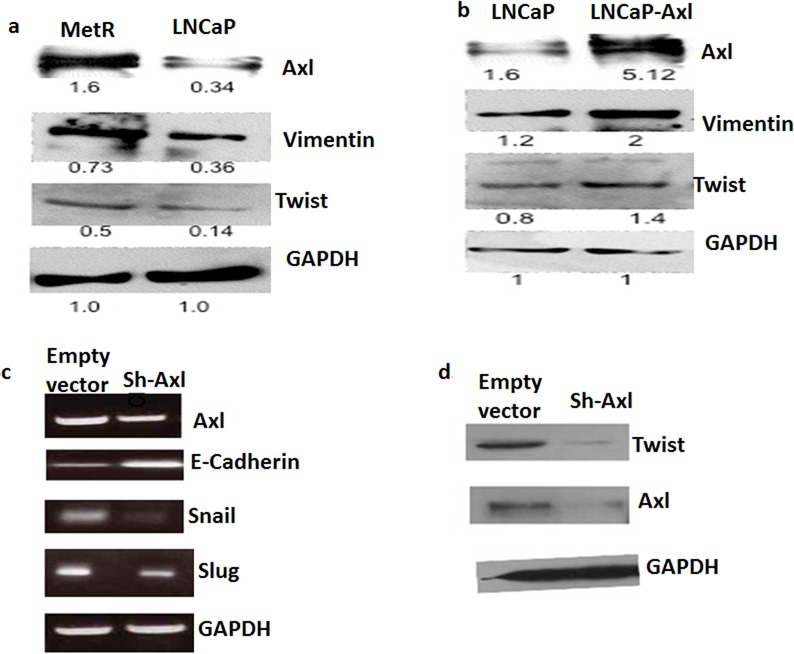
**a. MetR cells have increased expression of EMT markers:** 50ug of total protein extracted from MetR and LNCaP cells were loaded on SDS page gels. Western blots ere performed using anti-vimentin, anti-twist and anti-GAPDH antibodies. **b.** Axl overexpression in LNCaP cells caused an increase in expression of EMT proteins: Cells were either transfected with empty vector or with pcDNA-Axl plasmid. Total protein was extracted and western blotting was repeated as above to detect the protein levels in LNCaP and LNCaP-Axl cells. **c**, **d.** EMT markers are down-regulated when Axl is knocked down in Du145 cells: Axl was stably knockdown in Du145 cells using lentiviral transduction. Total RNA was extracted from Du145 and Du145-sh-Axl cells and RT-PCR was done with snail, slug, twist, E-cadherin and GAPDH primers. **d.** 50ug of total protein from Du145 and Du145-sh-Axl cells was loaded on a SDS page gel and western blotting was done using Axl, twist and GAPDH antibodies.

In several studies, Axl was shown to be involved in epithelial to mesenchymal transition (EMT), associated with drug resistance [20]. To show if Axl mediates metformin resistance by regulating EMT, we examined the expression of EMT proteins in the parenteral LNCaP and MetR cells as well as in Axl knock down and parenteral Du145 cells. E-cadherin is a marker for epithelial cells whereas vimentin, twist, snail and slug are markers for mesenchymal cells [21, 22]. In MetR cells and LNCaP cells transiently transfected with Axl, vimentin and twist expression were increased. (Figure 6a, 6b). In Axl knock out Du145 cells, E-cadherin expression was increased while twist, snail and slug were decreased (Figure 6c, 6d). These results, together with the morphologic changes and functional assays showing changes in the resistant cells (vide supra) indicate that resistance to metformin is associated with a phenotypic change of cells to EMT.

## DISCUSSION

Several studies have shown that diabetic patients who are metformin users have a lower incidence and mortality from cancer. For example, a large study followed patients who had used metformin and other diabetic drugs for 9.6 years [23]. Out of 1,300 patients, 289 had been treated with metformin, and 1064 were treated with another drug. Patients who were on metformin had a lower mortality due to cancer with an adjusted hazard ratio of 0.43 (95% CI-0.23-0.80). Another study [24] in which patients were treated with either metformin or sulfonylurea for a period of 5 to 5.5 years showed metformin users had lower mortality (3.5% vs 4.9% for the sulfonylurea cohort). These and other epidemiological studies have encouraged preclinical studies of metformin as treatment for patients with cancer. However, the mechanism of action of metformin as an anticancer drug is not completely understood. Some studies have indicated that metformin acts to inhibit cancer cells in both AMPK dependent and in an independent way. In an AMPK dependent way, it activates AMPK and inhibits mTOR [25] where as in an AMPK independent way, metformin inhibits mTORC1 in the absence of inhibition of AMPK and TSC1/2. REDD1 (regulated in development and DNA damage responses 1) is up regulated when cells are treated with metformin and it inhibits mTORC1 signaling in absence of AMPK and TSC1/2 activation. Another study demonstrated that metformin inhibits mTORC1 via Rag GTPAse. AMPK also regulates the expression and phosphorylation of p53 and studies in prostate cancer and colon cancer cells have shown that wild type p53 cells are more sensitive to metformin than p53 null cells [26, 27].

We show that castrate resistant prostate cancer cells such as Du145 and PC3 are less sensitive to metformin than androgen sensitive LNCaP cells. Thus to investigate further the mechanism of metformin inherent resistance as well as acquired resistance, and clues to its mechanism of action in prostate cancer, we developed metformin resistant LNCaP cells and performed a microarray gene expression analysis comparing sensitive and metformin resistant LNCaP cells. Genes encoding surface receptors or surface receptor ligands were up regulated, in particular *epiregulin* a ligand for EGFR receptor*s, Edil3,* epidermal growth factor-like repeats, discoidin I-like domains 3, *Annexin a2*, and *Axl receptor tyrosine kinase* were up regulated. A literature search on the up-regulated genes resulted in Axl attracting our attention. Axl belongs to the TAM family of tyrosine receptor kinases and plays a role in multiple processes viz. it inhibits apoptosis, increases proliferation and migration, suppresses inflammation [14] and is elevated in drug resistant cancers [16, 28-35]. Gas 6 binds to the extracellular domain of Axl and the Gas6/Axl complex dimerizes. Upon dimerization, tyrosine residues of the kinase domain are auto phosphorylated and the downstream signaling molecules PI3K and AKT are activated. Activated AKT then inhibits pro-apoptotic proteins [36]. Ligand independent dimerization and activation of Axl has also been observed. Homophilic binding of extracellular domain of Axl on the neighboring cells can also cause its activation [37]. Overexpression of Axl can also cause ligand independent auto activation of Axl [38], and pathophysiological conditions, e.g., increased ROS can also cause auto activation of Axl [39].

Increased levels of Axl have been shown to be associated with drug resistance. Examples are imatinib or nilotinib resistance in acute myelocytic leukemia [15, 29] and EGFR inhibitor associated resistance in non-small cell lung cancer [17]. However, the mechanism of this association is not completely understood. In a study published by Zhang et al. [16], increased expression of Axl in non-small cell lung cancer cells induced EMT and resistance to erlotinib, an EGFR inhibitor. Moreover, knock down of Axl reversed the acquired resistance. In prostate cancer cells, we see a similar phenotype; Du145 and PC-3 cells that overexpress Axl are intrinsically resistant to metformin and have an EMT phenotype and knock down of Axl renders the cells more sensitive to metformin. In non-small cell lung cancer, Axl expression is up-regulated in cells expressing mutant p53 and knockdown of endogenous mutant p53 led to the down regulation of Axl expression levels [40]. And as mentioned above, prostate cancer cells expressing wild type p53 have low expression of Axl and are sensitive to metformin. The relationship between levels of Axl and p53 mutant in cancer cells and their sensitivity to chemotherapy deserves further inquiry.

Currently there are many tyrosine kinase inhibitors approved that target multiple tyrosine kinases but are not specific to Axl. Carbozantinib, a multikinase inhibitor, is in phase 2 and 3 clinical trials against many different type of cancers. BMS-777607, another small molecule inhibitor against TAMs (Tyro3, Mer and Axl) is in phase I studies in solid tumors. R428 (BGB324) is a novel small molecule relatively specific Axl inhibitor that blocks auto-phosphorylation of Axl and inhibits the activation of AKT and SFK at low nanomolar concentrations [19]. In our studies, we show additive to synergistic effects with R428 and metformin in MetR and in Du145 cells. Reversal of drug resistance by down regulating or inhibition of Axl suggests that a combination of Axl inhibition with metformin as well as with other chemotherapeutic agents may be a novel therapeutic approach for the treatment of drug resistant cancers that overexpress Axl.

## MATERIALS AND METHODS

### Reagents

Metformin (1,1-Dimethylbiguanide hydrochloride) was purchased from Sigma Aldrich, RPMI media, R428 was purchased from Selleck Chemicals (Houston, Texas). FBS was purchased from GIBCO (Grand Island, NY). Antibodies for Axl and phospho-Axl were purchased from R&D systems (Minneapolis, MN). Primers for Axl were synthesized by Rutgers core facility (Piscataway, NJ); shRNA lentiviral constructs for Axl were purchased from Rutgers Core facility (Lentiviral library from Open Biosystems). MTS reagent was purchased from Promega (Madison, WI).

### Cell culture

Prostate cancer cells LNCaP, Du145, PC3 and CWR22 were cultured in RPMI media containing 10% FBS and 1% Penicillin-Streptomycin (Carlsbad, CA). Axl knock out Du145 cells were cultured in RPMI media with 1ug/ml puromycin.

### MTS cytotoxicity assay

MTS assay was used to determine the toxicity of metformin in prostate cancer cell lines. In brief, 5000 LNCaP, Du145 and PC3 cells were plated in 96 well dishes on Day1. The next day, metformin was added to the wells in increasing concentrations starting from 0.375mM to 50mM. Seventy-two hours after treatment with metformin, MTS reagent was added to the cells and the change in absorbance proportional to viability was measured at 490nm using SoftMax Pro (Molecular devices, Sunnyvale, CA). Percent survival is calculated by normalizing to untreated cells. For drug synergy experiments, combination index values (CI) values were calculated using CalcuSyn software CI>1 antagonism, CI = 1, additive, CI < 1, synergy [41]. The experiments were done in 96 well plate with 8 replicates. Standard deviation is shown in the graphs.

### Establishment of metformin resistant LNCaP cells

Two methods were used to generate metformin resistant LNCaP sublines; LNCaP cells were treated with either multiple exposures to the same IC50 dose (2.5mM), or starting with the IC50 concentration, and gradually increasing the metformin concentration (2.5mM to 5mM to 10mM). After 72 hours of treatment, drug was removed and fresh media was added and the cells were allowed to grow and attain maximum confluence. Once confluent, cells were then divided into two aliquots. Cells were then either treated again with the IC50 concentrations or treated with an IC90 concentration. This process was repeated for approximately 10-12 times until cells were resistant to metformin, as assayed by MTS.

### Gene expression microarray analysis

RNA from sensitive and resistant cells was isolated using TRIzol (Life technologies, Carlsbad, CA). Quality of total RNA (5 μg) was verified and processed for microarray analysis at the Laboratory of Molecular Technology, National Cancer Institute, Frederick, MD. Briefly, the RNA was reverse transcribed and hybridized to Affymetrix GeneChip Human Genome U133 Plus 2.0 array which is composed of more than 54,000 probe sets and 1,300,000 distinct oligonucleotides. It analyzes the expression level of over 47,000 transcripts and variants, including 38,500 well-characterized human genes. Three independent replicates for each of the experimental conditions were carried out and analyzed to control for intra-sample variation. Comparative analyses of expressed genes that were either down-regulated or up-regulated by >2-fold were carried out using the GeneSpring software available at the National Cancer Institute. The signal intensity were normalized using RMA (robust multiarray analysis) summarization and baseline transformation to median of all samples. Entities were filtered based on their signal intensity values. Hierarchical clustering was performed on filtered on signal intensity (>20.0), non-averaged, fold change >2. Gene ontology (GO) analysis was done using, fold change >2, p-value cutoff 0.1, as p-value cutoff 0.05 resulted in no significant GO groups. A fold change analysis (>10 fold) was performed to get list of top genes under/over represented in between the groups. Statistical analysis was performed using t-test (fold change >= 2, corrected p-value <= 0.05).

### Reverse transcription-PCR

Total RNA was extracted from parental LNCaP cells and metformin resistant LNCaP cells using TRIzol (Invitrogen, Carlsbad, CA) reagent. RT-PCR was performed using one step RT-PCR kit (Life Technologies, Carlsbad, CA) on equal amount of RNA from both the sets of cells using primers for Axl, Snail, Slug, E-cadherin and GAPDH primers. Primer sequence is as follows: Axl primers: Forward: 5′-AACCTTCAACTCCTGCCTTCTCGT-3′; Reverse: 5′-ACACATCGCTCTTGCTGGTGTAGA-3′; GAPDH primers: Forward: 5′-GAGTCAACGGATTTGGTCGT-3′; Reverse: 5′-TTGATTTTGGAGGGATCTCG-3′; Snail, forward 5′-ACCACTATGCCGCGCTCTT-3′ and reverse, 5′-GGTCGTAGGGCTGCTGGAA-3′; Slug, forward 5′-TGTTGCAGTGAGG-GCAAGAA-3′ and reverse 5′-GACCCTGGTTGCTTCAAGGA-3′; and E-cadherin: forward 5′-GTCAGTTCAGACTCCAGCCC-3′ and reverse 5′-AAATTCACTCTGCCCAGGACG-3′. PCR conditions were 94 °C for 15 s, 55 °C for 30 s, 72 °C for 1 min, and 30 cycles for each target. A final elongation step was carried out at 72 °C for 7 min. The PCR product was subjected to agarose gel electrophoresis and photographed using a Geldoc imager (Bio-Rad).

### Western blotting

Total protein was extracted from sensitive and metformin resistant cells using RIPA buffer supplemented with protease cocktail inhibitor (Roche; Nutley, NJ). 50ug of total protein was loaded on the SDS-page gel and protein was transferred on a nylon membrane. The transferred protein was then immunoblotted with antibodies against Axl, GAPDH, Twist and Vimentin.

### Axl overexpression and Axl knock down

LNCaP cells were transiently transfected with pcDNA-Axl plasmid using lipofectamine 2000. 48 hours after transfection, cells were harvested and western blot was done to confirm the overexpression of Axl protein. Axl was knocked out using pGIPZ lentiviral plasmid for shAxl (Rutgers DNA Core facility, Piscataway, NJ). In brief, Du145 cells were transfected with the pGIPZ-sh-Axl plasmid using lipofectamine 2000 (Life Technologies, Carlsbad, CA). 48 hours after transfection, cells were selected with 1ug/ml puromycin for the transfected cells. The cells were also monitored under fluorescence microscope for the transfection efficiency. Western blot was done to confirm the knock down of Axl. In MetR cells, Axl was knocked down using si-RNA oligos for Axl (Sigma Aldrich; St Louis, MO). Oligos for si-Axl were transirently transfected with Lipofectamine 2000.

## SUPPLEMENTARY TABLE



## Abbreviations

Axl RTK: Axl Receptor Tyrosine Kinase
MetR: Metformin Resistance
EMT: Epithelial to Mesenchymal transition
